# Interleukin-4 Expressed By Neoplastic Cells Provokes an Anti-Metastatic Myeloid Immune Response

**Published:** 2015-05-31

**Authors:** Connie S. Zhang, Hyeyeon Kim, Graeme Mullins, Kathrin Tyryshkin, David P. LeBrun, Bruce E. Elliott, Peter A. Greer

**Affiliations:** Department of Pathology and Molecular Medicine, Queen’s University, Division of Cancer Biology and Genetics, Cancer Research Institute, Kingston, Ontario, K7L 3N6, Canada

**Keywords:** Orthotopic engraftment, Metastasis, Interleukin-4, M2 polarization, Tumor associated macrophages

## Abstract

**Objective:**

Interleukin-4 (IL-4) can induce macrophages to undergo alternative activation and polarize toward an M2-like or wound healing phenotype. Tumor associated macrophages (TAMs) are thought to assume M2-like properties, and it has been suggested they promote tumor growth and metastasis through effects on the tumor stroma, including extracelluar matrix remodeling and angiogenesis. IL-4 also promotes macrophage survival and formation of multinucleated giant cells, which have enhanced phagocytic behavior. This study was designed to explore the effect of cancer cell derived IL-4 on the tumor immune stroma and metastasis.

**Methods:**

The metastatic mouse mammary carcinoma cell line AC2M2 was transduced with control or IL-4 encoding retroviruses and employed in orthotopic engraftment models. Tumor growth and metastasis were assessed. The cellular composition and biomarker expression of tumors were examined by immunohistochemical staining and flow cytometry; the transcriptome of the immune stroma was analyzed by nanoString based transcript quantitation; and *in vivo* and *in vitro* interactions between cancer cells and macrophages were assessed by flow cytometry and co-culture with video-time lapse microscopy, respectively.

**Results:**

Unexpectedly, tumors from IL-4 expressing AC2M2 engrafted cells grew at reduced rates, and most surprising, they lost all metastatic potential relative to tumors from control AC2M2 cells. Myeloid cell numbers were not increased in IL-4 expressing tumors, but their expression of the M2 marker arginase I was elevated. Transcriptome analysis revealed an immune signature consistent with IL-4 induced M2 polarization of the tumor microenvironment and a generalized increase in myeloid involvement in the tumor stroma. Flow cytometry analysis indicated enhanced cancer cell phagocytosis by TAMs from IL-4 expressing tumors, and co-culture studies showed that IL-4 expressing cancer cells supported the survival and promoted the *in vitro* phagocytic behavior of macrophages.

**Conclusions:**

Although M2-like TAMs have been linked to enhanced tumorigenesis, this study shows that IL-4 production by cancer cells is associated with suppressed tumor growth and loss of metastatic potential as well as enhanced phagocytic behavior of TAMs.

## Introduction

IL-4 was first described as B-cell growth factor [1]; and subsequently shown to promote immunoglobulin class switching of B cells [2]. Besides its effects on B-cells, IL-4 has broad functions in multiple cell types of hematopoietic and non-hematopoietic origin. It skews the differentiation of naïve T (Th0) cells toward Th2 helper T cells, at the expense of Th1 differentiation, and promotes Th2 production of other cytokines including IL-5, IL-10 and IL-13 [3]. IL-4 induces vascular endothelial cells to express vascular cell adhesion molecule-1 (VCAM-1), which recruits T cells, eosinophils and basophils to sites of inflammation [4]. IL-4 is a survival factor, protecting lymphocytes and myeloid progenitors from apoptosis [5,6]; and it is also a potent macrophage fusion factor, capable of inducing macrophages to form large multinucleated giant cells, which are central players in inflammatory responses to foreign materials including implants [7,8]. In contrast to M1-like classical macrophage activation by LPS and IFNγ, IL-4 promotes alternative M2 macrophage activation [9–11]. Tumor associated macrophages (TAMs) are enriched for cells with this M2 polarized phenotype, which have been suggested to promote tumor cell growth, invasion and metastasis by secreting paracrine-acting and extracellular matrix remodeling factors including EGF, MMP and VEGF [12]. Upon ligand binding, the IL-4 receptor activates Jak1 and Jak3 kinases, which activate Stat6, promoting its translocation into the nucleus to initiate the transcription of IL-4 target genes [13–15]. In TAMs, those target genes include arginase I, which is a recognized marker for M2 polarized macrophages.

The role of IL-4 in cancer is unclear. Contradictory findings have been reported that suggest IL-4 could either enhance or inhibit cancer cell proliferation *in vitro* depending on the cancer cell type [16–21]. IL-4 has been shown to protect against apoptosis in cultured prostate, breast, thyroid, and bladder tumor cell lines [16,20]. Clinical trials of injected recombinant human IL-4 in patients with renal cell carcinoma, chronic lymphocytic leukemia, or non-Hodgkin’s lymphoma have been unsuccessful [22–24]. However, other studies have shown that malignant tumor cells genetically engineered to produce IL-4 displayed potent anti-tumor effects *in vivo*, suggesting the potential use of IL-4 secreting tumor cells as tumor vaccines for treating patients with advanced cancers [25–28]. While these preclinical studies documented significant reduction in tumor growth at the injection sites of IL-4 expressing tumor cells, the effect of IL-4 on the metastatic spread of these tumors was not assessed. In order to explore this question, we engineered the metastatic AC2M2 mouse carcinoma cell line to express IL-4 and assessed them using an orthotopic mammary gland engraftment model. We found that mice engrafted with IL4-AC2M2 cells grew tumors at a significantly reduced rates as compared to mice engrafted with empty vector (EV) transduced AC2M2 cells. Even more striking, mice bearing IL4-AC2M2 tumors developed no metastasis, while EV-AC2M2 tumors consistently produced extensive metastasis. While the numbers of tumor associated myeloid cells were unchanged, IL4-AC2M2 tumors displayed a 10-fold increase in strongly staining arginase I positive cells. This is consistent with *in vitro* observations showing that conditioned medium from IL-4 transduced tumor cells activated the Jak-Stat pathway and arginase I expression in the tumor cells. Using a nanoString immunology probe set to assess the transcriptome in these tumors, we observed an immune signature consistent with an M2 polarized myeloid tumor immune stroma. Flow cytometry assessment of these tumors revealed evidence of increased cancer cell phagocytosis by TAMs, and co-culture experiments suggested that cancer cell derived IL-4 promoted macrophage survival and phagocytic activity. These observations support the exploration of using IL-4 in therapeutic strategies, such as tumor vaccines or oncolytic viruses, and suggest that cancer cell derived IL-4 may promote cancer cell killing by myeloid cells.

## Materials and Methods

### Cell lines

AC2M2, a highly metastatic basal-like murine mammary carcinoma cell line that arose spontaneously in a retired CBA/J breeder strain [29] and HEK-293T (ATCC) cells were routinely cultured in DMEM (Invitrogen) containing 10% FBS (Sigma), 2mM L-glutamine, and 2mM antibiotic-antimycotics (AA; Invitrogen) in a 5% CO2 humidified incubator at 37°C. Retroviruses or lentiviruses were produced by co-transfection of HEK-293T cells with retroviral packaging plasmid (Φ-NX-ECOpac for retroviruses and pCMV-ΔR8.91 and pMD.2G for lentiviruses) along with the proviral pMSCVpuro retroviral plasmid (Clontech) or the pWPI lentiviral plasmid (kindly provided by Didier Trono). AC2M2 cells were transduced with lentiviruses expressing green fluorescence protein (GFP) and high GFP expressing cells were selected by fluorescence activated cell sorting. GFP-expressing AC2M2 cells were transduced with pMSCVpuro retroviruses encoding recombinant murine IL-4 (IL4) or the empty vector control (EV). BMA3.1A7 (BMA) cells, a macrophage cell line derived from C57BL/6 mice [30], were grown in complete Roswell Park Memorial Institute (RPMI) culture medium containing 5% FBS, 2mM L-glutamine, and 2mM AA.

### ELISA

The Mouse IL-4 ELISA Ready-SET-Go!^®^ kit (eBioscience) was used according to the manufacture’s recommended protocol. Samples included culture medium collected from HEK- 293T cells transfected with retroviral packaging plasmids and EV- or IL4-proviral pMSCV plasmids or non-transfected cells, as well as parental, EV- or IL4-retrovirus transduced AC2M2 cells. 10X, 100X, and 1000X fold dilutions were assayed in triplicate.

### Cell proliferation assay

4 × 10^4^ EV- or IL4-AC2M2 cells were plated in triplicate on 6-well plates. At 4 hours post plating, and at 24 hour intervals thereafter for 5 days, cells were collected with trypsin/EDTA, and counted using a Z1 Coulter Particle Counter (Beckman).

### *In vitro* assessment of tumor cell secreted IL-4 biological activity

To confirm biological activity of tumor cell-derived recombinant IL-4, conditioned media collected from EV-AC2M2 or IL4-AC2M2 cell cultures were added to BMA cell monolayers at 5, 20, 50 or 100% of total culture medium. Other BMA cells were treated with 2.5, 5 or 10 μg/mL recombinant mouse IL-4 (rIL-4) as positive controls, or media alone as a negative control. Cell lysates were prepared at 24 hours and used for immunoblotting analysis.

### Immunoblotting analysis

Lysates from control mammary glands or tumor-bearing glands were prepared in RIPA buffer (10mM Tris pH 7.2, 158mM NaCl, 1mM EDTA, 0.1% SDS, 1% sodium deoxycholate, 1% Triton 100 with 10 μg/mL aprotinin, 10 μg/mL leupeptin, 100 μM sodium orthovanadate, 100 μM 150 phenylmethylsulfonyl fluoride) using a Ultra-Turrax T25 homogenizer (Terochem Scientific). For cell cultures, cells were washed with ice-cold PBS containing 100 μM vanadate, and then lysed with RIPA buffer for 20min. The remaining procedures have been described in detail previously [31,32]. Briefly, lysates were centrifuged at 13,000 × g for 15 min at 4°C to obtain the soluble fractions. After protein quantification, samples were diluted with SDS sample buffer and heated for 5 min at 95°C. Proteins were then resolved by SDS-PAGE and transferred to membranes using a semi-dry transfer apparatus (Bio-Rad). Membranes were blocked with either 5% skimmed milk or 5% BSA for 1 hr, and incubated overnight with primary antibody at 4°C. After washing in TBST, membranes were incubated with the appropriate secondary antibody for 1 hr at room temperature, and proteins were detected using enhanced chemiluminescence reagents (NEN Life Science Products). Antibodies used in immunoblotting included anti-arginase I (BD Bioscience), anti-Stat6 (Upstate), anti-tubulin (Sigma), anti-phospho-Stat6, anti-phospho-Jak1, anti-Jak1 and anti-phospho-Jak3, which were all purchased from Cell Signaling.

### Mice

Mice used in this study included nude (NCr-Foxn1^nu/nu^; Taconic) and BALB/c Rag2^−/−^; IL2Rγc^−/−^ double-knockout (kindly provided by Dr. M. Ito, Central Institute for Experimental Animals, Kawasaki, Japan). Mice were housed in the Queen’s University Animal Care Facility and procedures were carried out according to the guidelines of the Canadian Council on Animal Care, with the approval of the institutional animal care committee.

### Orthotopic mouse engraftment model

After subcutaneous injection of meloxicam (2 mg/kg), female age-matched nude or Rag2^−/−^; IL2Rγc^−/−^ mice were anesthetized by isoflurane inhalation and injected with 7,500 GFP expressing EV- or IL4-AC2M2 cells into the fourth inguinal mammary gland. Tumor growth was assessed by caliper measurements. For metastasis studies, tumors were resected by recovery surgery either at equal times post engraftment, or when tumors had reached equal tumor volume endpoints. These mice were kept alive for additional 12 days to allow metastatic lesions to grow to easily detectable sizes. Mice were then euthanized, lungs were dissected and GFP expressing metastatic nodules were imaged and quantitated as described previously [31].

### NanoString analysis of tumor transcriptomes

EV- or IL4-AC2M2 cells were engrafted into the fourth inguinal mammary gland of Rag2^−/−^; 180 IL2Rγc^−/−^ mice as described above, and tumors were resected at equal volumes (~800 mm^3^). The duration of tumor growth before resection were 18 days for EV tumors and 32 days for IL4- expressing tumors. RNA was extracted from tumors using Trizol reagent (Sigma) according to manufacturer instructions. In biological triplicates, 100 ng of RNA was assayed using the nCounter Mouse Immunology CodeSet with added custom genes according to manufacturer protocol. Results were normalized and analyzed using the NanoStriDE software package with default settings [33].

### Peritoneal lavage

After mice were euthanized by deep isoflurane inhalation and cervical dislocation, 5 mL of prewarmed lavage media [RPMI1640 (Invitrogen), 10 mM HEPES, 5mM EDTA, 10 U/mL heparin, 1% AA and 50 μM α-monothioglycerol] were injected into the peritoneum, followed by gentle message of the abdomen and collection of the lavage media. Cells were isolated by centrifugation and resuspended in 5mL of erythrocyte lysis buffer (154mM NH_4_Cl, 10mM KHCO_3_, 100 μM EDTA) for 5 min on ice to lyse red blood cells. The remaining cells were then resuspended in peritoneal macrophage medium [RPMI1640 (Invitrogen), 5% FBS, 1mM HEPES, 1% AA, 2mM glutamine and 50 μM α-monothioglycerol], plated and incubated in a 5% CO_2_ humidified incubator at 37°C. After 2 hours, non-adherent cells were washed off with PBS, and adherent cells (peritoneal macrophages) were used for subsequent experiments.

### Macrophage-tumor cell co-culture

Peritoneal macrophages were labeled with CellTracker^™^ Orange CMRA according to the manufactures protocol (Invitrogen), and then trypsinized and seeded onto a 6-well plate containing gelatin-coated coverslips. After 24 hours of co-cultivation with either EV-AC2M2 or IL4-AC2M2 GFP-expressing cells, coverslips were mounted onto glass slides with Mowiol (Calbiochem) and imaged using spinning-disc fluorescence microscopy.

For video time-lapse analysis, CellTracker^™^ 204 Orange CMRA labeled peritoneal macrophages were co-cultured with GFP-expressing AC2M2 cells on 8 well μ-slide (Idbi) for 24 hours, and then images were taken every 5 minutes using spinning-disc fluorescence microscopy for 18 hours. Movies were made using MetaMorph^®^ microscopy automation and Image analysis software. Further quantitative analysis were done using ImagePro software.

### Flow cytometry

Control EV- and IL4-AC2M2 tumor-bearing mammary glands were harvested from Rag2^−/−^; IL2Rγc^−/−^ mice. These glands were digested in tissue digestion buffer and single cell suspensions were prepared as described in detail previously [31]. Samples were stained with 0.1 μg/mL PE-conjugated rat anti-mouse F4/80 antibody (Caltag) for macrophages, washed with ice-cold 1% BSA/PBS, and analyzed by flow cytometry.

### Immunohistochemical staining

Five μm sections of formalin fixed paraffin embedded control EV- and IL4- AC2M2 tumor bearing mammary glands were stained with arginase I for macrophages, CD11b for myeloid cells, cleaved caspase-3 for cell apoptosis, and Ki67 for cell proliferation using an automated Ventana staining system Discovery XT. High resolution images were scanned and quantified using an Aperio ImageScope and Software system (Aperio, Vista, CA).

### Statistics

All statistical analysis was done by using GraphPad Prism software. All error bars represent standard error of the mean. P values were calculated by Student’s *t*-test analysis. Data sets with P ≤ 0.05 were considered significant.

## Results

### Tumor cell derived IL-4 induced biological effects on macrophages *in vitro*

GFP-expressing AC2M2 mouse mammary carcinoma cells were transduced with empty vector control (EV-) or mouse IL-4 expressing (IL4-) retroviruses. To confirm IL-4 expression, conditioned medium (CM) from parental, EV-AC2M2 and IL4-AC2M2 cell cultures were analyzed by ELISA. An IL-4 concentration of 3.2 ng/mL was measured in IL4-AC2M2 cell CM, while no detectable IL-4 was apparent in parental or EV-AC2M2 cell CM.

**Figure 1:**
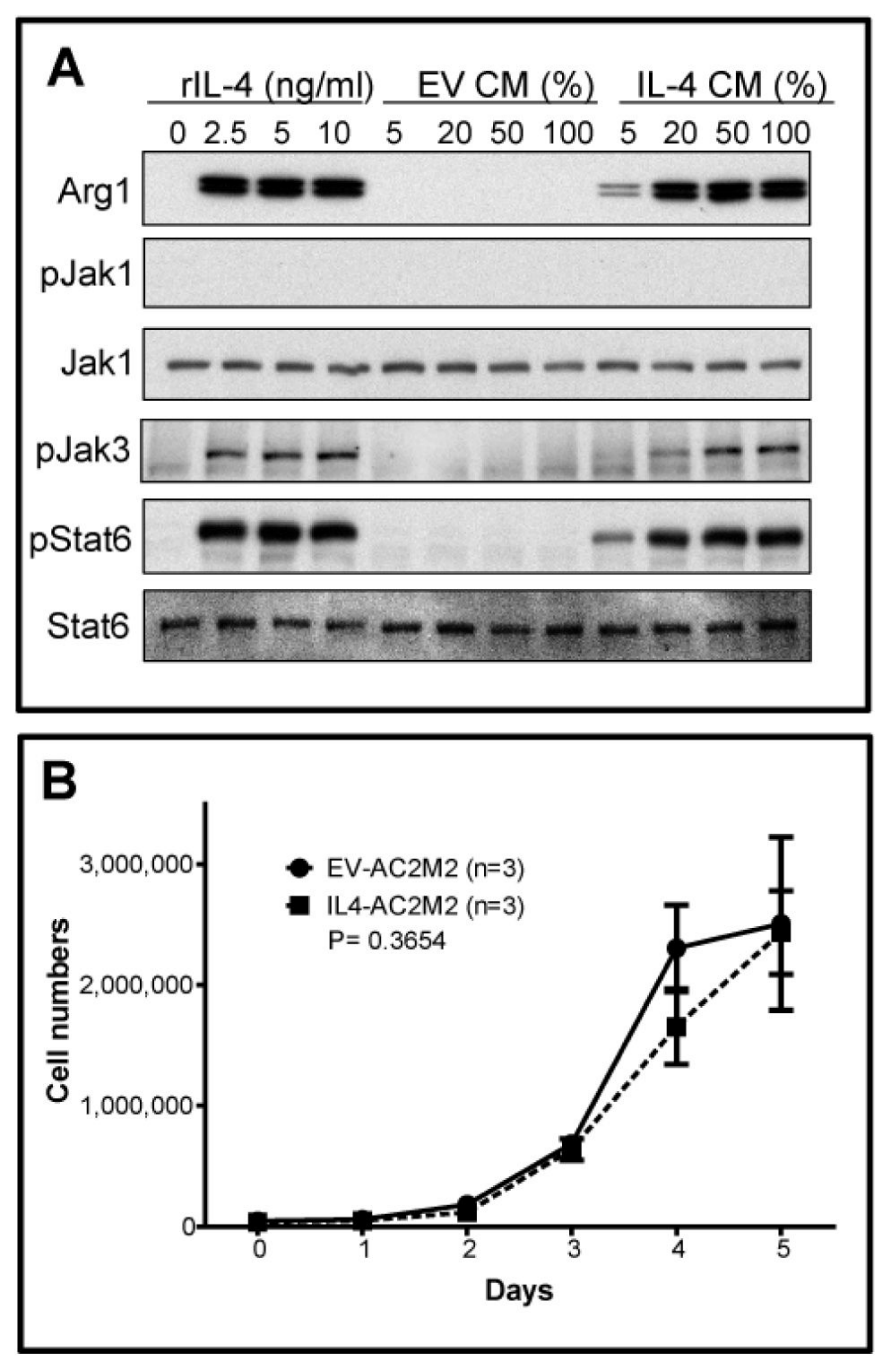
IL-4 produced by AC2M2 cells promotes M2-like macrophage activation but does not promote autocrine proliferation effects. A: Conditioned medium (CM) collected from EV-AC2M2 (EV CM) or IL4-AC2M2 (IL-4 CM) cells was added to BMA cell cultures at increasing CM concentrations of 5%, 20%, 50% or 100%, and cultured for 24 hours. BMA cells were also challenged with 2.5, 5 or 10 ng/ml recombinant mouse IL-4 (rIL-4) for positive controls. Lysates were analyzed by immunoblotting for macrophage polarization using anti-arginase I (Arg1) antibody, and Jak/Stat activation using anti-phospho-Jak1 (pJak1), anti-phospho-Jak3 (pJak3) and anti-phospho-Stat6 (pStat6) antibodies. B: EV-AC2M2 or IL4-AC2M2 cells were seeded in triplicate (n=3) on 6-well plates. Four hours later or at the indicated days post plating, cells were trypsinized, and cell numbers were counted using Z1 Coulter Particle Counter. No significant difference was observed in growth rates *in vitro* between EV-AC2M2 and IL4-AC2M2 cells (P=0.3654).

IL-4 is a Th2 type cytokine reported to be capable of activating the Jak-Stat pathway and inducing an alternative pathway of macrophage activation and polarization into an M2-like, or wound healing phenotype [34–36]. To confirm that recombinant IL-4 secreted by IL4-AC2M2 cells had biological activity, BMA mouse macrophages were cultured for 24 hrs with increasing concentrations of conditioned medium from either EV-AC2M2 or IL4-AC2M2 cells, or with recombinant mouse IL-4 (rIL-4) at concentrations of 2.5, 5 or 10 ng/ml, and then assessed for activation of the Jak-Stat pathway and expression of the M2 marker arginase I (Arg1). Conditioned media from IL4-AC2M2 elicited a dose responsive phosphorylation of Jak-3 and Stat-6, as well as induction of Arg1 expression that mirrored the response of recombinant IL-4, while conditioned media from EV-AC2M2 had no effects on this pathway (Figure 1A). Jak-1 in BMA cells was not detectably activated by IL-4. These observations confirmed that IL4-AC2M2 cells produce biologically active IL-4 capable of stimulating the Jak-Stat pathway in macrophages and promoting expression of the M2 marker, Arg1.

While different types of cancer cell lines have been reported to undergo a range of responses to IL-4, including pro-survival, pro-apoptotic or mitogenic behaviours [16,17,19–21], we found that EV-AC2M2 and IL4-AC2M2 cells grew at indistinguishable rates (P=0.3654, Figure 1B), suggesting that they do not express 4 receptors and therefore display no apparent autocrine effects associated with IL-4 expression.

### IL-4 expression from engrafted cancer cells is associated with suppressed tumor growth and loss of metastatic potential

**Figure 2:**
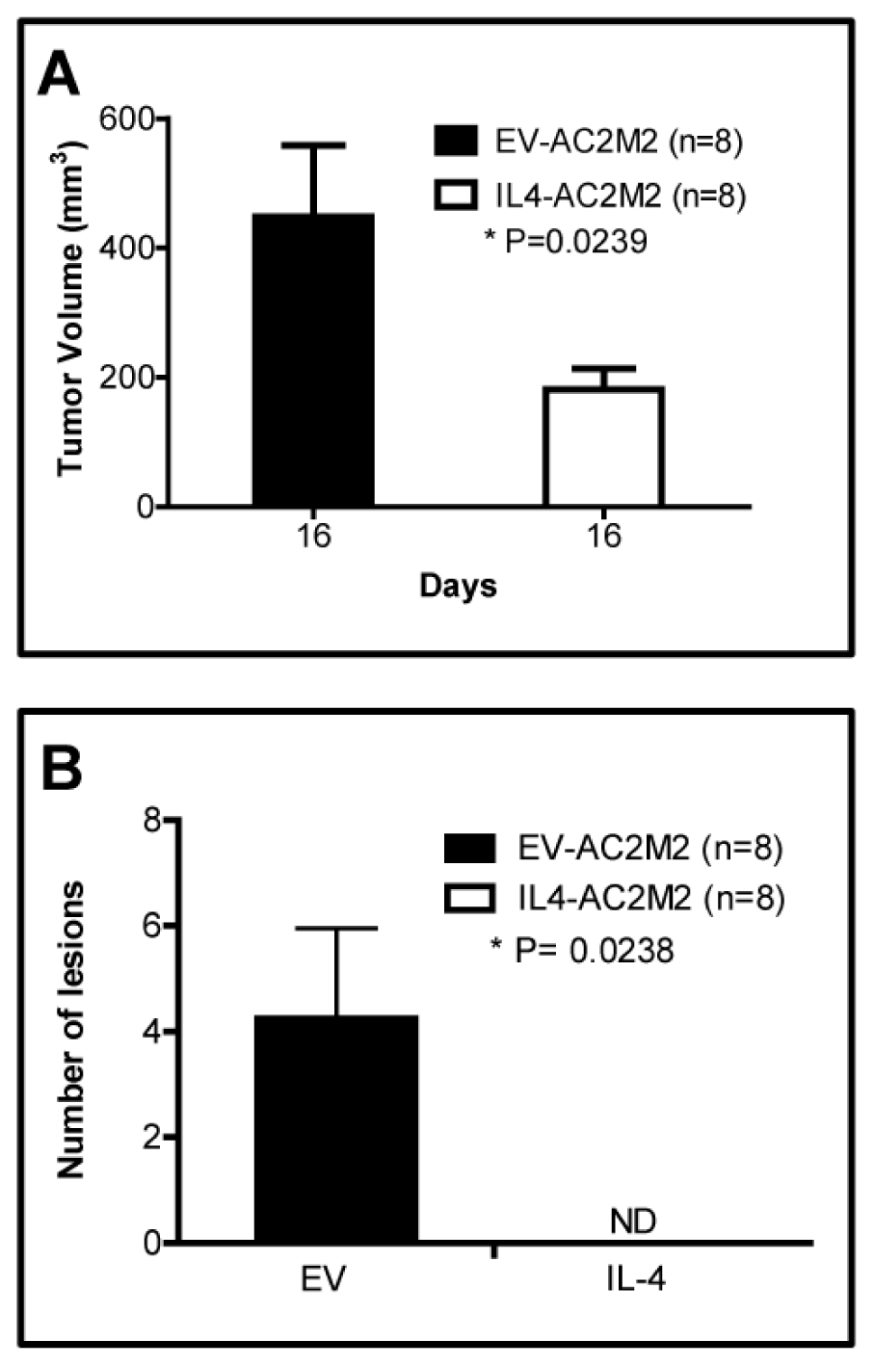
IL-4 expression from cancer cells suppressed tumor grow and metastasis in a nude mouse orthotopic engraftment mode. A: EV-AC2M2 or IL4-AC2M2 cells were engrafted into the fourth mammary glands of nude mice and tumor volume was measured with calipers on day 16 post-engraftment. Tumors were significantly smaller in mice engrafted with IL-4 expressing AC2M2 tumor cells (IL4-AC2M2) compared to those with control tumor cells (EV-AC2M2) (P=0.0239; n=8 for both groups). B: Tumors were resected by recovery surgery on day 16 post-engraftment. On day 28 post-engraftment, mice were sacrificed and lungs were removed for biophotonic imaging of metastatic lesions. 38% (3 of 8) of the lungs harvested from control tumor-bearing mice (EV-AC2M2) harbored detectable metastasis while none (0 of 8) of the lungs from IL-4 expressing tumor-bearing mice (IL4-AC2M2) displayed lung metastasis (P=0.0238; n=8 for each group; ND, not detectable). Data shown are representative of two independent experiments.

We next explored the *in vivo* effects of cancer cell-derived IL-4 on tumorigenesis by orthotopically engrafting GFP-expressing EV-AC2M2 or IL4-AC2M2 cells into mouse mammary glands and comparing tumor growth and metastasis. In athymic nude mice, IL4-AC2M2 tumor volumes on day 16 post-engraftment were significantly reduced relative to control EV-AC2M2 tumors (P=0.0239, Figure 2A).

Tumor bearing mammary glands were resected on day 16 by recovery surgery, and metastasis was assessed 12 days later. This “resect and wait” approach has been found to promote the growth of metastatic nodules at distant sites to sizes that are easily detectable [37]. The AC2M2 cell line was established as a lung-homing metastatic variant line [29]. Lung metastatic nodules were detected in 3 of 8 nude mice engrafted with EV-AC2M2 cells. In contrast, no lung metastasis was observed in 8 tumor bearing mice that were engrafted with IL4-AC2M2 cells (P=0.0238, Figure 2B, Supplementary Figure 1).

**Figure 3:**
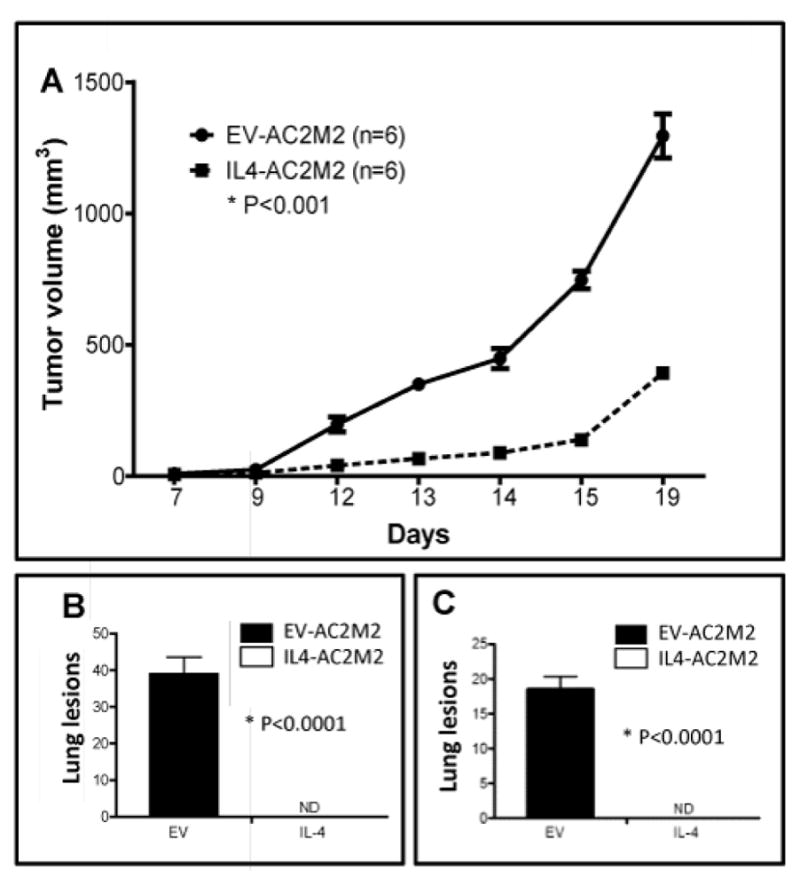
IL-4 expression from cancer cells suppressed tumor grow and metastasis in a Rag2^−/−^; IL2Rγc^−/−^ mouse orthotopic engraftment mode. A: EV-AC2M2 or IL4-AC2M2 cells were engrafted into the fourth mammary glands of Rag2^−/−^; IL2Rγc^−/−^ mice and tumor growth was assessed over a period of 19 days. Tumor growth rate of IL-4 expressing tumors (IL4-AC2M2) was significantly slower as compared to control tumors (EV-AC2M2) (P<0.001; n=6 for each group). B: Tumors were resected by recovery surgery on day 19 post-engraftment. On day 31 post-engraftment, 12 days after tumor resection, mice were sacrificed and lungs were removed for biophotonic imaging of metastatic lesions. 100% (6 of 6) of the lungs harvested from control tumor-bearing mice (EV-AC2M2) harbored detectable metastasis while none (0 of 6) of the lungs from IL-4 expressing tumor-bearing mice (IL4-AC2M2) displayed lung metastasis (P<0.0001; n=6 for each group; ND, not detectable). C: Tumors were resected on day 14 or day 19 post-engraftment for EV-AC2M2 and IL4-AC2M2, respectively, by recovery surgery when they had achieved a volume of ~500mm3. Twelve days after tumor resection mice were sacrificed and lungs were removed for biophotonic imaging of metastatic lesions. 100% (6 of 6) of the lungs harvested from control tumor-bearing mice (EV-AC2M2) harbored detectable metastasis while none (0 of 6) of the lungs from IL-4 expressing tumor-bearing mice (IL4-AC2M2) displayed lung metastasis (P<0.0001; n=6 for each group; ND, not detectable). Data shown are representative of four independent experiments.

Since the Foxn1^nu/nu^ athymic nude mouse model lacks functional lymphoid cells but retains natural killer (NK) cells, which are known to play roles in blocking metastasis, we were curious to see how IL-4 might influence tumorigenesis in Rag2^−/−^; IL2Rγc^−/−^ mice, which lack T cells, B cells and NK cells. Again, we observed slower IL4-AC2M2 tumor growth at the orthotopic engraftment site over the 19 day observation period, relative to EV-AC2M2 tumors (P<0.001, Figure 3A). Primary tumors were resected on day 19 and mice were kept alive for additional 12 days, then euthanized and assessed for metastatic lesions. Lungs from all mice engrafted with EV-AC2M2 cells displayed extensive metastasis, with an average of 40 lung lesions. In contrast, no metastasis was observed in any of the tumor-bearing mice engrafted with IL4-AC2M2 cells (P<0.0001, n=6, Figure 3B, Supplementary Figure 2).

Since control tumors were approximately 3 times larger than IL-4 expressing tumors at the time of resection (1300 vs 400 mm^3^, Figure 3A), we wondered if some of the difference in metastasis frequency might be due to the differences in tumor sizes at the time of resection. To address this, the engraftment experiment with Rag2^−/−^; IL2Rγc^−/−^ mice was repeated as before, except tumor-bearing glands from each cohort were resected when tumors had grown to an average size of 500 mm^3^. Five additional days were required for IL4-AC2M2 tumors to achieve this size (Figure 3A). Both groups of mice were kept alive for the same length of time (12 days) after tumor resection, and lung metastasis was assessed as described above. Again, lungs harvested from all mice engrafted with EV-AC2M2 cells displayed metastasis with an average of 18 metastatic nodules per lung. In contrast, no metastasis was observed in any mice from the IL4-AC2M2 cohort (P<0.0001, n=6, Figure 3C, Supplementary Figure 3). These observations argue that tumor size is not responsible for the difference in metastatic potential.

### IL-4 production by cancer cells was associated with M2 polarization and anti-tumorigenic behaviour of tumor associated myeloid cells

**Figure 4:**
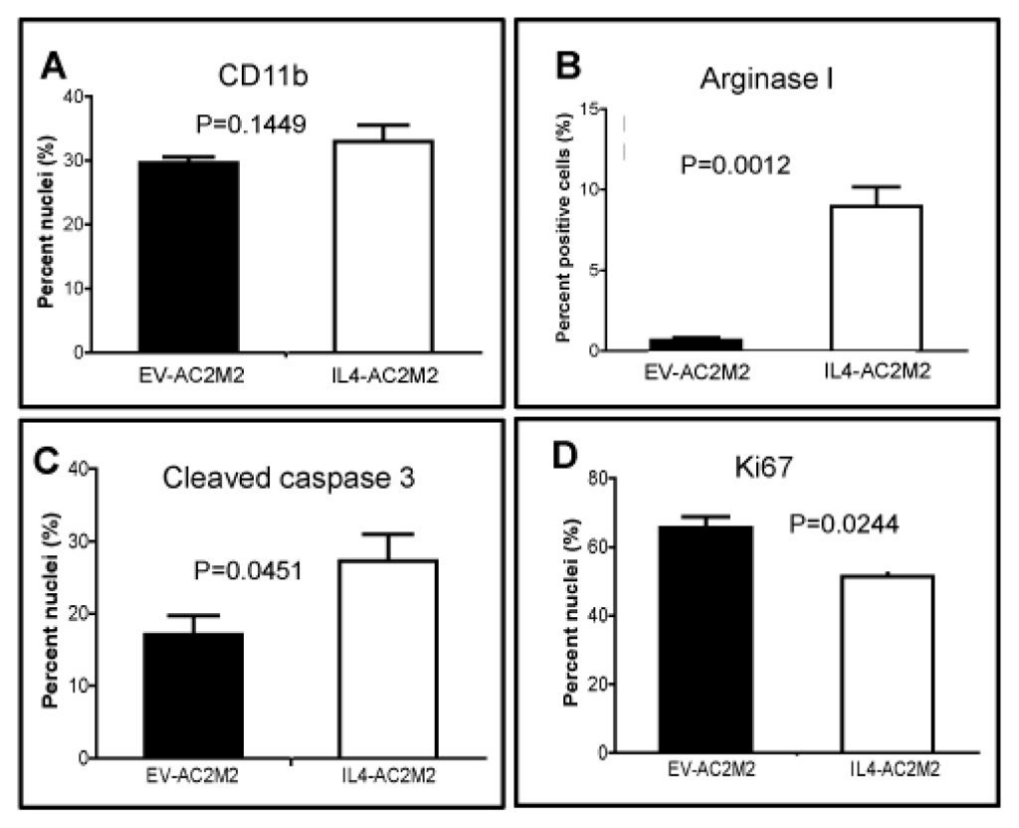
IL-4 expressing tumors contain evidence of M2-polarized myeloid cells, reduced proliferation and increased apoptosis. EV-AC2M2 or IL4-AC2M2 tumors were resected at 500 mm^3^ volumes from engrafted Rag2^−/−^; IL2Rγc^−/−^ mice, fixed with paraformaldehyde and embedded in paraffin (n=3 for each cohort). Five μm sections were stained using an automated Ventana system with the indicated antibodies. Quantitation was carried out using Aperio image analysis software. A: CD11b, P=0.1449; B: arginase I, P=0.0012; C: cleaved caspase 3, P=0.0451; and D: Ki67, P=0.0244.

We next explored the effect of IL-4 from cancer cells on the composition and phenotypes of tumor-associated immune cells. Since Rag2^−/−^; IL2Rγc^−/−^ mice lack T cells, B cells and NK cells, our *in vivo* analysis was restricted to cells of the myeloid lineage. IHC staining of sections from tumor-bearing glands revealed similar levels of CD11b^+^ cells in both cohorts, indicating that IL-4 did not cause a significant increase in the number of tumor associated myeloid cells (Figure 4A, P=0.1449, Supplementary Figure 4). However, the frequency of Arg1 staining cells was elevated ~10 fold in the IL4-AC2M2 cohort, suggesting that IL-4 was influencing the polarization of these tumor associated myeloid cells (Figure 4B, P=0.0012, Supplementary Figure 5). This IHC result was validated by immunoblotting analysis of tumor bearing glands, which showed abundant levels of Arg1 protein in IL4-AC2M2 tumors, but no detectable Arg1 in EV-AC2M2 tumors or normal mammary glands (Supplementary Figure 6). These observations were also consistent with *in vitro* experiments showing that conditioned media from IL4-AC2M2 cells promoted activation of the Jak-Stat pathway and induction of the M2 marker Arg1 in cultured macrophages (Figure 1A). This ~10 fold increase in Arg1^+^ cells in the absence of a significant increase in CD11b^+^ cells suggests that IL-4 produced by tumor cells promoted M2 polarization of tumor associated myeloid cells *in vivo*.

### IL-4 induces an M2 polarized immune signature in the tumor stromal

**Figure 5:**
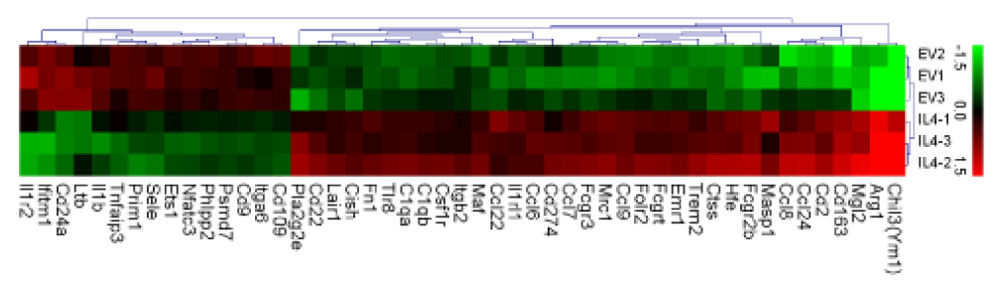
Heat map of genes with significant changes of expression in IL4 vs EV tumors. In biological triplicates, 100 ng of total tumor RNA was assayed using the nCounter Mouse Immunology CodeSet with added custom genes according to manufacturer protocol. Results were normalized and analyzed using NanoStriDE software package with default settings. Genes with significantly changed expression were analyzed with hierarchical clustering by Euclidean distance using MultiExperiment Viewer v.49 (MeV). Data was transformed by natural logarithm and normalized to the average expression across all samples.

IL-4 production from cancer cells is expected to have pleotropic effects on the tumor stroma through influencing the recruitment of immune cells and gene expression within immune and other cell types in the tumor microenvironment. In order to gain a broader perspective on the effects of IL-4 on the tumor microenvironment we assessed expression of a panel of 691 immune genes using nanoString-based transcript quantification. Genes showing the highest level of upregulation in IL-4 tumors included IL-4 itself (480 fold), but also a number of genes that are known to be upregulated in association with macrophage M2 polarization, such as Ym1 (54 fold), Arg 1 (22 fold), Mgl2 (9 fold), CD163 (7 fold), Ccl24 (5 fold) and Mrc1 (4 fold) (Sup Table 1). Analysis of these data with Ingenuity IPA predicted significant changes in several biological functions consistent with an activated myeloid response in IL4 tumors, including increased viability of myeloid cells, increased cytolysis, increased migration of antigen presenting cells, and decreased cell death of granulocytes (Figure 5, Supplementary Table 1).

### IL-4 from cancer cells promotes macrophage survival and phagocytosis

**Figure 6:**
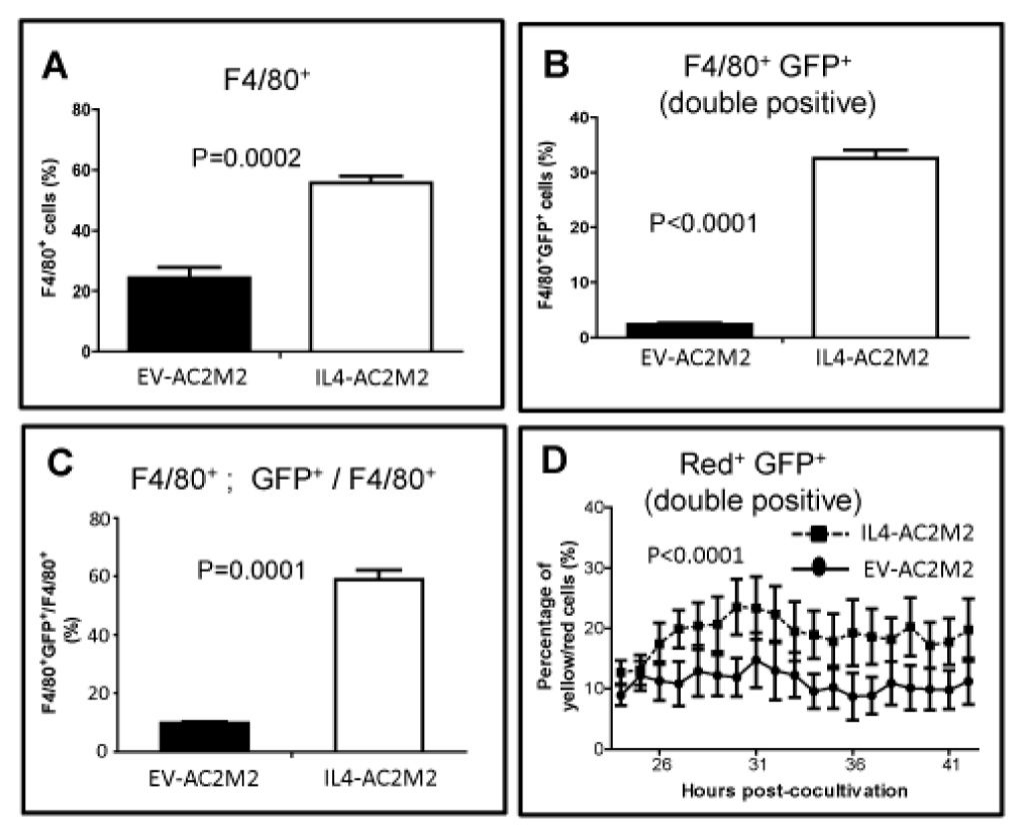
Cancer cell derived IL-4 promotes phagocytic behavior in tumor associated macrophages *in vivo* and cultured macrophages *in vitro*. GPF expressing EV-AC2M2 or IL4-AC2M2 tumors were resected at 500mm^3^ volumes from engrafted Rag2^−/−^; IL2Rγc^−/−^ mice. Single cell suspensions were stained with PE-conjugated F4/80 antibody to identify macrophages and analyzed by flow cytometry (n=4 for each cohort). A: F4/80^+^ macrophages were elevated in IL4-AC2M2 relative to EV-AC2M2 tumors (P=0.0002). B: F4/80^+^ GFP+ double positive cells were elevated in IL4-AC2M2 relative to EV-AC2M2 tumors (P<0.0001). C: The proportion of F4/80^+^ cells which were double positive for F4/80 and GFP was elevated in IL4- AC2M2 relative to EV-AC2M2 tumors (P=0.0001). D: Orange cell tracker dye labeled peritoneal macrophages (red) were co-cultured with either GFP-expressing EV-AC2M2 or IL4-AC2M2 cells for 24 hours. Nine random fields were then selected from each co-culture and imaged every 5 minutes for 18 hours by video time-lapse microscopy. Numbers of yellow cells and red macrophages were quantified using ImagePro software. Higher percentages of yellow cells were found in IL4-AC2M2 co-cultures (P<0.0001; n=9 for both groups).

IHC staining for cleaved caspase 3 showed higher levels of apoptosis in IL4-AC2M2 tumors (P=0.0451, Figure 4C, Supplementary Figure 7), and staining for Ki67 showed less proliferation in the IL4-AC2M2 tumors (P=0.0244, Figure 4D, Supplementary Figure 8). This was consistent with the observed slower rates of IL4-AC2M2 tumor growth, but did not provide direct evidence for immune cell killing of cancer cells. IL-4 has been shown to promote macrophage survival and induce fusion to form multinucleate giant cells which have greater phagocytic capability [7,8,38,39]. While IHC analysis of CD11b expressing cells in tumor sections did not reveal significant differences in the frequency of tumor-associated myeloid cells (Figure 4A), the increase in Arg 1 positive cells (Figure 4B) and the nanoString transcriptomic analysis suggested a significant effect of IL-4 on the phenotype of tumor associated myeloid cells. Flow cytometry analysis of tumors showed elevated numbers of F4/80^+^ macrophages in the IL4-AC2M2 tumors (P=0.0002, Figure 6A). Furthermore, IL4-AC2M2 tumors contained ~10 fold higher numbers of F4/80^+^; GFP^+^ (double positive) cells (P=0.0001, Figure 6B, Supplementary Figure 9). This suggested that tumor associated macrophages were engaged in phagocytosis of the GFP^+^ cancer cells to a higher degree in the IL4-AC2M2 tumors. This was further supported by the observation that F4/80^+^ cells in IL4-AC2M2 tumors were ~6 times more likely to be double positive for F4/80 and GFP (P=0.0001, Figure 6C, Supplementary Figure 9).

In order to directly observe tumor cell macrophage interactions, GFP expressing EVAC2M2 or IL4-AC2M2 cells (green) were co-cultured with cell tracker dye labeled peritoneal macrophages (red) for 24 hours, and then imaged by fluorescence microscopy (Supplementary Figure 10). Smaller, presumably dying macrophages predominated in co-cultures with control EV-AC2M2 cells, while larger viable macrophages, as well as macrophage clusters (possible fused macrophages) were apparent in IL4-AC2M2 co-cultures. More yellow cells were also seen in IL4-AC2M2 co-cultures, indicative of macrophage-tumor cell fusion or phagocytosis events. To observe these interactions in real time, we performed video time lapse analysis of these co-cultures for a further 18 hours. More dynamic macrophage-tumor cell interactions were apparent in co-cultures with IL4-AC2M2 cells, and image analysis along this 18 hour time course revealed significantly more yellow cells in the IL4-AC2M2 co-cultures consistent with a higher degree of phagocytic or cell fusion behavior of these macrophages (P<0.0001, Figure 6D).

## Discussion

This study provides evidence that IL-4 could provide therapeutic benefit in breast cancer treatment by suppressing both tumor growth and metastasis, in part through an immune modulatory effect on tumor-associated myeloid cells. Previous reports have suggested that IL-4 could either promote or inhibit cancer cell proliferation *in vitro* by exerting effects through binding to IL-4R expressed on cancer cells [17,19,21,40]. While a direct mechanism might come into play in the context of some tumors, it is unlikely to have been involved in the model system used here because we observed no difference in cell growth rates between AC2M2 breast carcinoma cells transduced with either empty vector control or IL-4. Rather than affecting tumor growth in an autocrine fashion, tumor-cell derived IL-4 may have effects on tumorigenesis and metastasis by modulating cells in the tumor niche in a paracrine manner. IL-4 has been reported to have broad functions in multiple cell types of hematopoietic origin [2,5,7,8,10,34,38,41–45]. When we performed mouse orthotopic engraftment experiments with AC2M2 breast carcinoma cells expressing empty vector control or IL-4 in immune-compromised athymic nude mice or Rag2^−/−^; IL2Rγc^−/−^ double-knockout mice, we observed the most pronounced differences in tumorigenesis and metastasis in Rag2^−/−^; IL2Rγc^−/−^ mice. Since these mice have no T-cells, B-cells or NK cells, we surmise that the differences in tumor growth and metastasis are most likely associated with cells of the myeloid lineage in the tumor microenvironment.

The concept of classic versus alternative macrophage activation, as well as the role of IL-4 in alternative macrophage activation, has been extensively described in recent years [45–47]. Classically, activated inflammatory M1-like macrophages are induced by IFNγ, LPS, TNF-α, and GM-CSF; and they mediate resistance to microbial infections as well as anti-tumorigenic properties [47]. IL-4 induces “alternative” activation of M2-like macrophages; and in response to other cytokines, including TGFβ, IL-10, and CSF-1, this macrophage type has been reported to acquire properties that enhance tumor cell growth, invasion and metastasis [45,46]. This is thought to involve secretion of extracellular matrix remodeling factors including MMPs and TGFβ, as well as growth factors such as EGF and VEGF [11,48,49].

When the BMA macrophage cell line was stimulated *in vitro* with mouse recombinant IL-4 or IL-4 containing conditioned medium from IL4-AC2M2 cells, the Jak-3/Stat-6 pathway was activated and they expressed arginase I, a common M2 macrophage marker, in a dose dependent manner. This observation was consistent with the expectation that IL-4 would be able to induce M2-like polarization of macrophages. This M2-like macrophage activation by cancer cell derived IL-4 was also apparent *in vivo* when these IL4-AC2M2 cells were used to generate orthotopic tumors in mice, as shown by increased arginase I expression in tumors, seen by both IHC and immunoblotting analysis. This was also supported by nanoString analysis which revealed upregulation of a number of classical M2 markers. Ingenuity pathway analysis of these nanoString data suggested the IL-4 tumors had a significantly higher degree of myeloid immune involvement. Collectively, these observations argue that cancer cell derived IL-4 can induce M2 macrophage activation *in vitro* as well as in the tumor microenvironment *in vivo*.

Since alternatively activated M2 macrophages are implicated in promoting tumor growth and metastasis [45], we were surprised to see less aggressive tumor growth in mice engrafted with IL-4 expressing AC2M2 cells. Even more surprising, there was a complete block in metastasis associated with cancer cell derived IL-4 expression. In addition to its survival enhancing role, IL-4 has been reported to act as a potent macrophage fusion factor, inducing macrophages to form large multinucleated giant cells which have enhanced phagocytic capability [7,8,38,39]. Thus, we speculated that tumor cell-derived IL-4 would promote increased numbers of tumor-associated macrophages, and that these would be more phagocytic. This was supported by flow cytometry analysis which detected ~2 times more F4/80^+^ macrophages in IL-4 expressing tumor stroma. More importantly, these F4/80^+^ macrophages were ~6 times more likely to be GFP^+^, which demonstrated a higher degree of phagocytic behavior toward cancer cells. This was also supported by *in vitro* co-culture analysis that showed a correlation between cancer cell IL-4 expression and enhanced macrophage survival, fusion and phagocytic behavior. This finding suggests that tumor-derived IL-4 promoted the recruitment and/or survival of more F4/80^+^ macrophages into the tumor microenvironment; but perhaps more importantly, that these macrophages are more phagocytic toward these IL-4 expressing cancer cells. While analysis of Ki67 and cleaved (active) caspase 3 in tumors was consistent with the reduced rates of tumor growth at the orthotopic site, the more exciting observation was the complete abolishment of metastasis in this highly metastatic engraftment model.

Several studies have used malignant tumor cells genetically engineered to produce IL-4 and shown potent anti-tumor effects *in vivo* associated with inhibiting primary tumor burden [25–28]. Our studies support an inhibitory effect of tumor-derived IL-4 on primary tumor growth, but perhaps even more significantly, they showed a dramatic effect of IL-4 in eliminating distant metastasis. To our knowledge, this effect on metastasis has not previously been described. Our observations suggest that reduced primary tumor growth and suppression of metastasis is associated with M2-like TAMs induced by cancer cell-derived IL-4. Additional studies using macrophage depletion and syngeneic engraftment models with gene knockouts that compromise the development or function of specific immune cells are needed to further elucidate the biologic basis of this IL-4 effect. It will also be important to determine how cancer cell derived IL4 influences other immune cell types including other myeloid cells as well as lymphoid cells, and their interactions with one another in the context of innate and adaptive immunity, as well as immune suppression. Fully immune competent syngeneic models will be essential for these future studies. These findings support the use of IL-4 as a potential therapeutic agent for patients with metastatic cancers. Clinical trials have shown that systemic administration of IL-4 plus GM-CSF was able to enhance dendritic cell number and their functions in cancer patients [50]. Both IL-4 and GM-CSF have shown promise as immune modulatory cargoes in the context of tumor vaccine and oncolytic virus approaches [25,51–53]. In summary, this study provides novel evidence that IL-4 expression by tumor cells might attenuate their metastatic potential by provoking direct myeloid cell-mediated killing.

## Supplementary Material
