# Supplementary material for: Interleukin-4 Expressed By Neoplastic Cells Provokes an Anti-Metastatic Myeloid Immune Response
Source: J Clin Cell Immunol. Author manuscript; Available in PMC 2016 Aug 23. (PMC4995104)
Supplement: Supplementary file 1 [file NIHMS5885-supplement-supplement_1.pdf]

Supplementary Figure 1

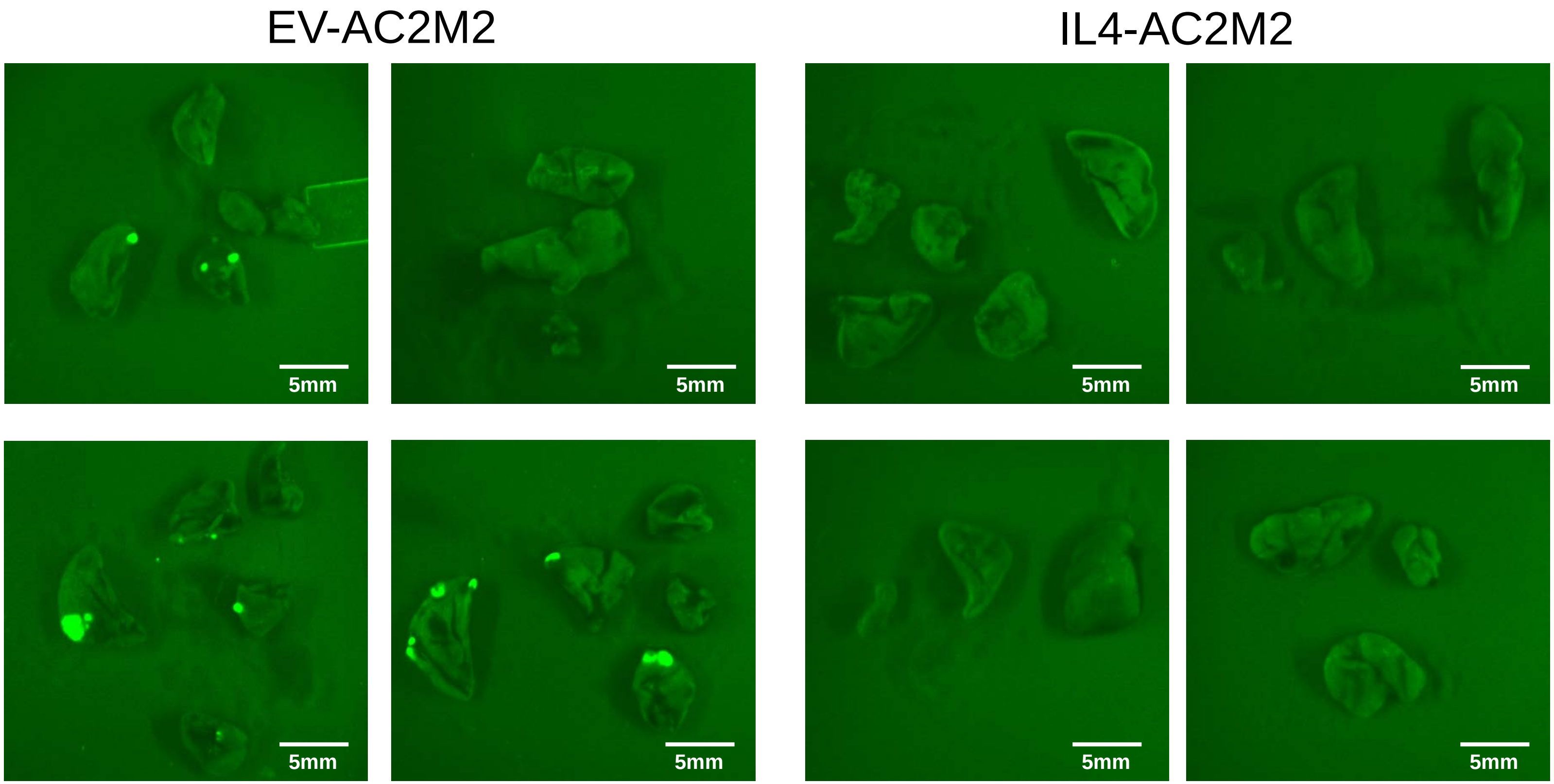

Supplementary Figure 1. Reduced lung metastasis in nude mice with IL-4 expressing tumors. 7,500 GFP-expressing EV-AC2M2 or IL4-AC2M2 cells were engrafted into the fourth mammary glands of nude mice and allowed to grow for 16 days. Tumors were then removed to promote tumor growth at metastatic sites and lungs were removed for biophotonic imaging of metastatic lesions 12 days later. 38% (3 of 8) of the lungs harvested from control tumor-bearing mice (EV-AC2M2) harbored detectable metastasis while none (0 of 8) of the lungs from IL-4 expressing tumor-bearing mice (IL4-AC2M2) displayed lung metastasis (P=0.0238; n=8 for each group). Photographs are representative images of lungs from four different mice of each group.

Supplementary Figure 2

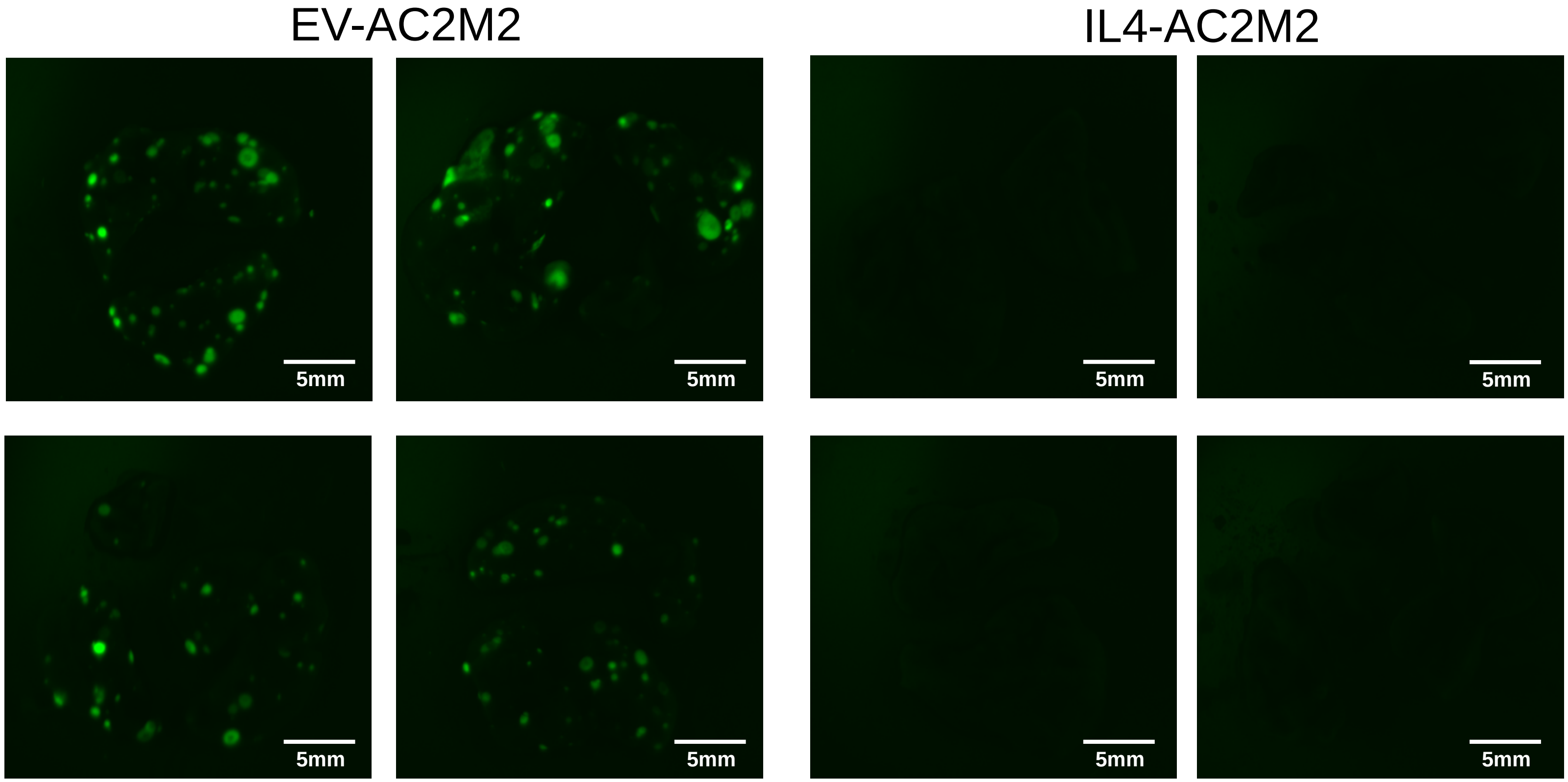

Supplementary Figure 2. Complete elimination of lung metastasis in Rag2-/- ; IL2Rγc-/- mice bearing IL-4 expressing tumors. 7,500 GFP-expressing EV-AC2M2 and IL4-AC2M2 cells were engrafted into the fourth mammary glands of Rag2-/- ; IL2Rγc-/- mice and allowed to grow for 19 days. Tumors were then removed to promote tumor growth at metastatic sites and lungs were removed for biophotonic imaging of metastatic lesions 12 days later. 100% (6 of 6) of the lungs harvested from control tumor-bearing mice (EV-AC2M2) harbored detectable metastatic lesions while none (0 of 6) of the lungs from IL-4 expressing tumor-bearing mice (IL4-AC2M2) displayed lung metastasis ($P<0.0001$; $n=6$ for each group). Photographs are representative images of lungs from four different mice of each group.

Supplementary Figure 3

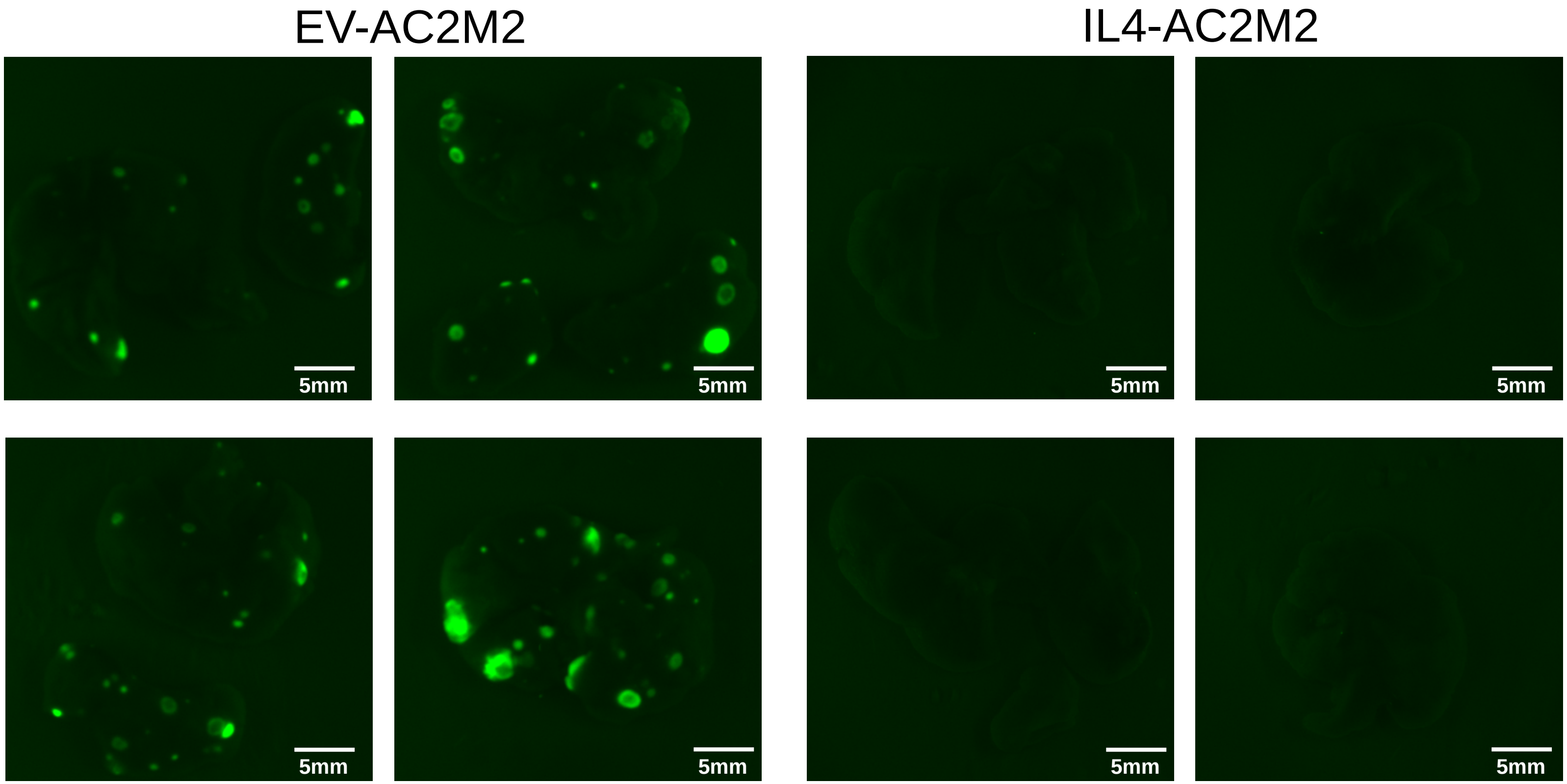

Supplementary Figure 3. Complete elimination of lung metastasis in Rag2-/- ; IL2Rγc-/- mice bearing IL-4 expressing tumors when control and IL-4 expressing tumors were removed at the same size. 7,500 GFP-expressing EV-AC2M2 and IL4-AC2M2 cells were engrafted into the fourth mammary glands of Rag2-/- ; IL2Rγc-/- mice. Tumors were removed at the same size (~500mm^3) from both groups. Mice were kept alive for 12 days after tumor resection surgery. Mice were then euthanized and lungs were dissected for biophotonic imaging of metastatic lesions. 100% (6 of 6) of the lungs harvested from control tumor-bearing mice (EV-AC2M2) harbored detectable metastatic lesions while none (0 of 6) of the lungs from IL-4 expressing tumor-bearing mice (IL4-AC2M2) displayed lung metastasis ($P<0.0001$; n=6 for each group). Photographs are representative images of lungs from four different mice of each group.

Supplementary Figure 4

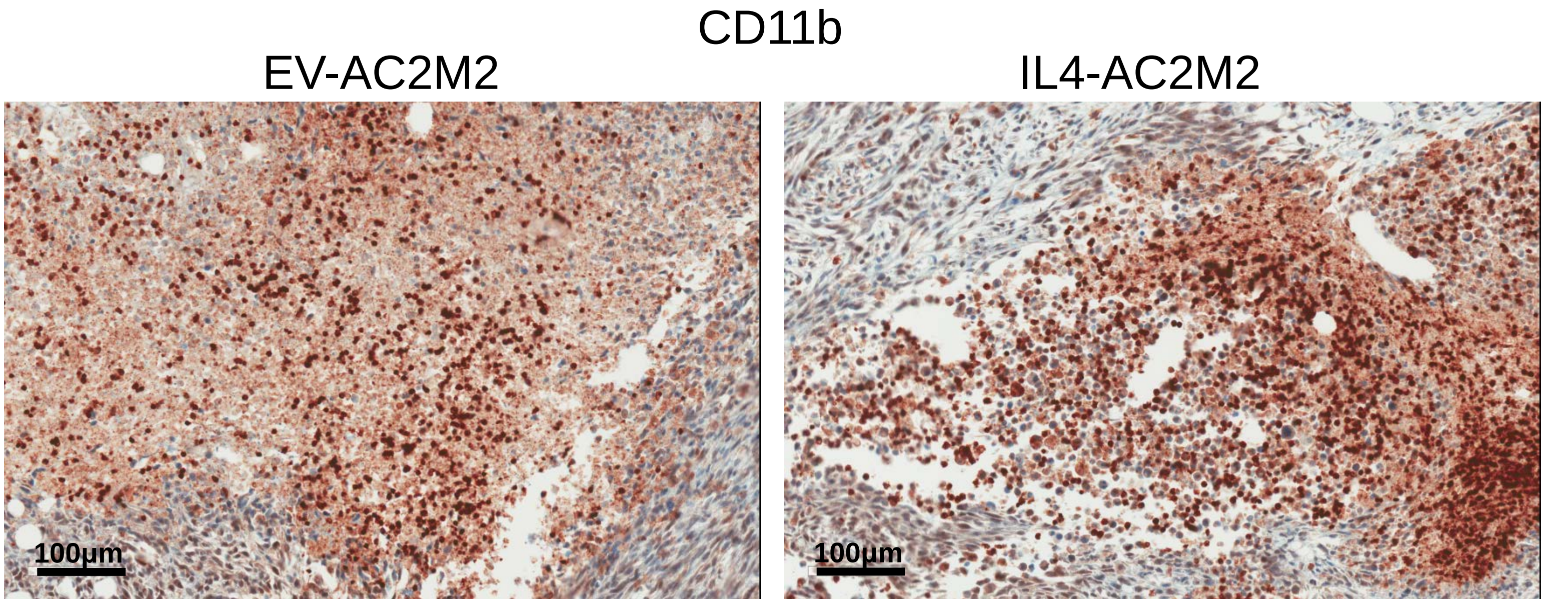

Supplementary Figure 4. CD11b staining of myeloid cells in EV-AC2M2 and IL4-AC2M2 tumors. Five micron thick tissue sections of control (EV-AC2M2) or IL-4 expressing (IL4-AC2M2) tumors were stained with antibodies to CD11b using a Ventana automated staining system and the number of CD11b-expressing cells were enumerated using Aperio software. The fraction of CD11b positive cells were not significantly different between the two cohorts (P=0.1449; n=3 for both groups).

Supplementary Figure 5

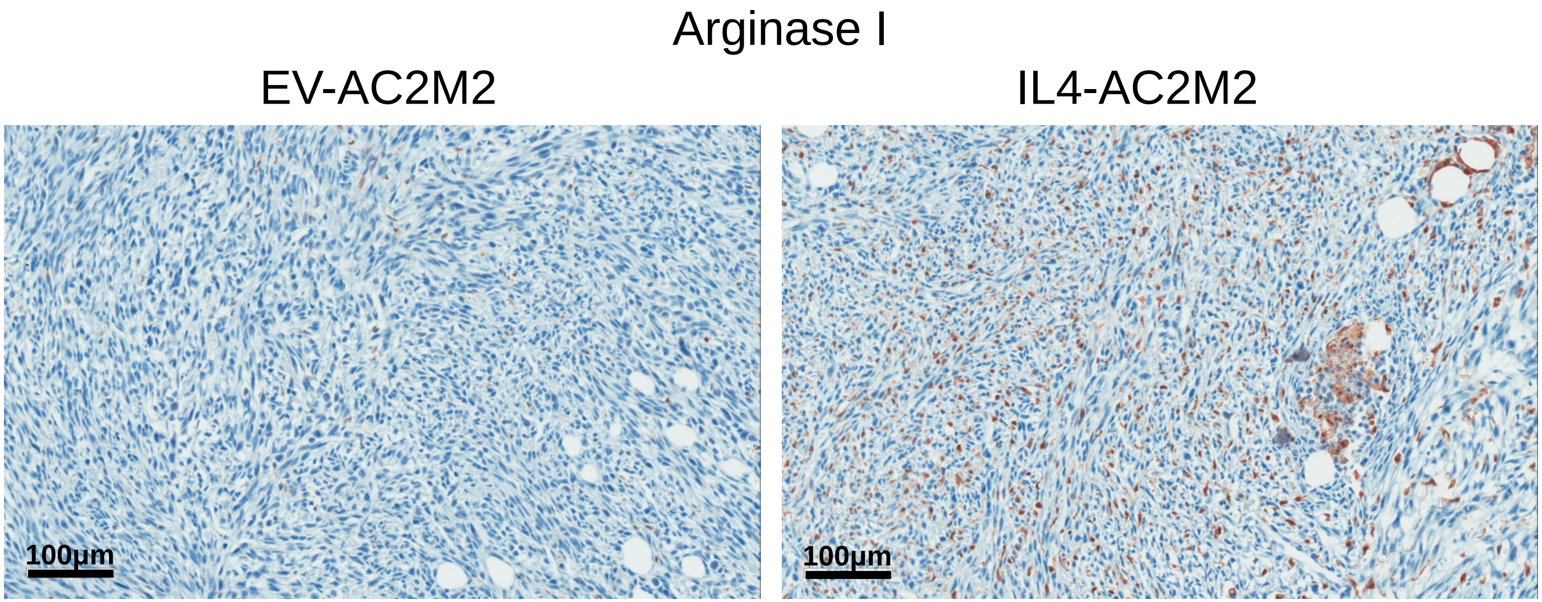

Supplementary Figure 5. Arginase I staining of cells in EV-AC2M2 and IL4-AC2M2 tumors. Five μm thick tissue sections of control (EV-AC2M2) or IL-4 expressing (IL4-AC2M2) tumors were stained with antibodies to Arginase I using a Ventana automated staining system and immuno-reactive cells were enumerated using Aperio software. The fraction of Arginase I positive cells was ~10 fold increased in IL4-AC2M2 tumors relative to control EV-AC2M2 tumors (P=0.0012; n=3 for both groups).

Supplementary Figure 6

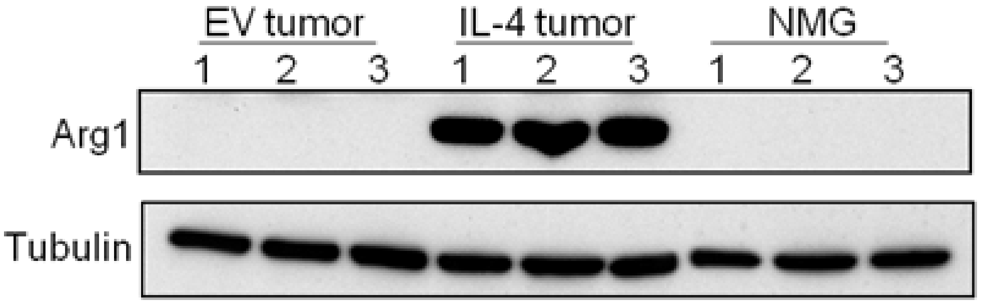

Supplementary Figure 6. High levels of arginase 1 expression in mammary glands bearing IL-4 expressing tumors. 7,500 GFP-expressing EV-AC2M2 or IL4-AC2M2 cells were engrafted into the fourth mammary glands of Rag2$^{-/-}$; IL2Rγc$^{-/-}$ mice and tumorigenesis was monitored. Control (EV tumor) and IL-4 (IL-4 tumor) expressing tumor-bearing mammary glands were harvested on day 13 and 16 respectively when tumors reached the same size (~500mm^3) and bisected for either immunoblotting analysis or immunohistochemical tissue staining. Lysates were made from 3 EV tumors, 3 IL-4 tumors and 3 normal mammary glands (NMG), and blotted with anti-arginase 1 (Arg1) antibody. High levels of Arg1 expression were detected in all three IL-4 expressing tumor-bearing mammary gland samples, whereas control tumor-containing mammary glands and normal mammary glands had no detectable Arg1 expression. Anti-tubulin immunoblotting demonatrated normalized protein loading.

Supplementary Figure 7

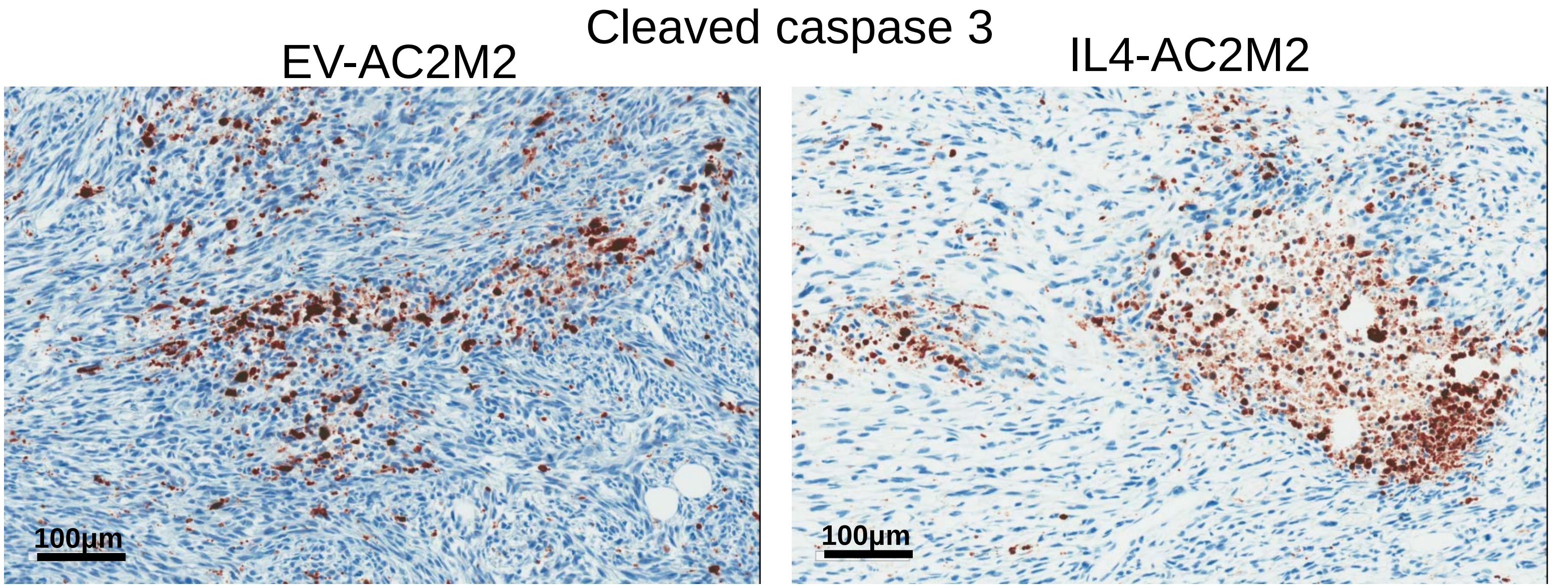

Supplementary Figure 7. Cleaved caspase 3 staining of apoptotic cells in EV-AC2M2 and IL4-AC2M2 tumors. Five micron thick tissue sections of control (EV-AC2M2) or IL-4 expressing (IL4-AC2M2) tumors were stained with antibodies to cleaved (active) caspase 3 using a Ventana automated staining system and immuno-reactive cells were enumerated using Aperio software. The fraction of cleaved caspase 3 positive apoptotic cells was significantly increased in IL4-AC2M2 tumors (P=0.0451; n=3 for both groups).

Supplementary Figure 8

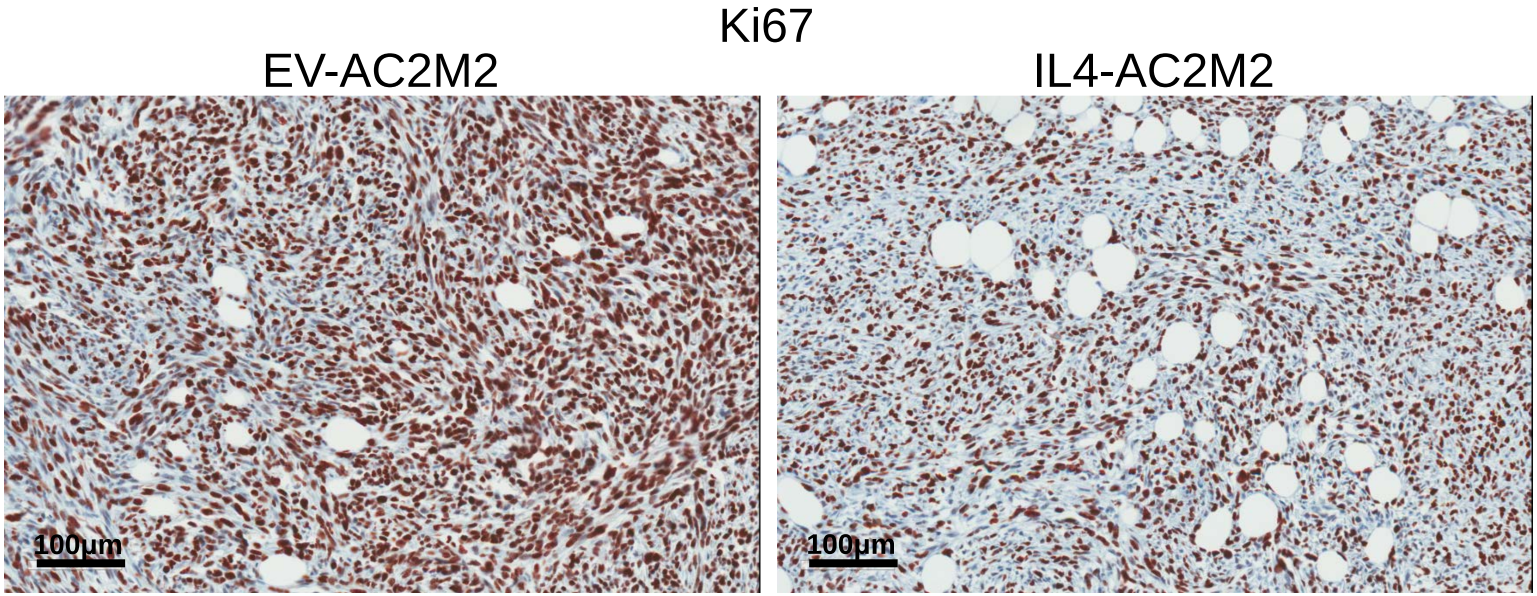

Supplementary Figure 8. Ki67 staining of mitotic cells in EV-AC2M2 and IL4-AC2M2 tumors. Five micron thick tissue sections of control (EV-AC2M2) or IL-4 expressing (IL4-AC2M2) tumors were stained with antibodies to Ki67 using a Ventana automated staining system and immuno-reactive cells were enumerated using Aperio software. The fraction of Ki67 positive mitotic cells was significantly decreased in IL4-AC2M2 tumors relative to control EV-AC2M2 tumors (P=0.0244; n=3 for both groups).

Supplementary Figure 9

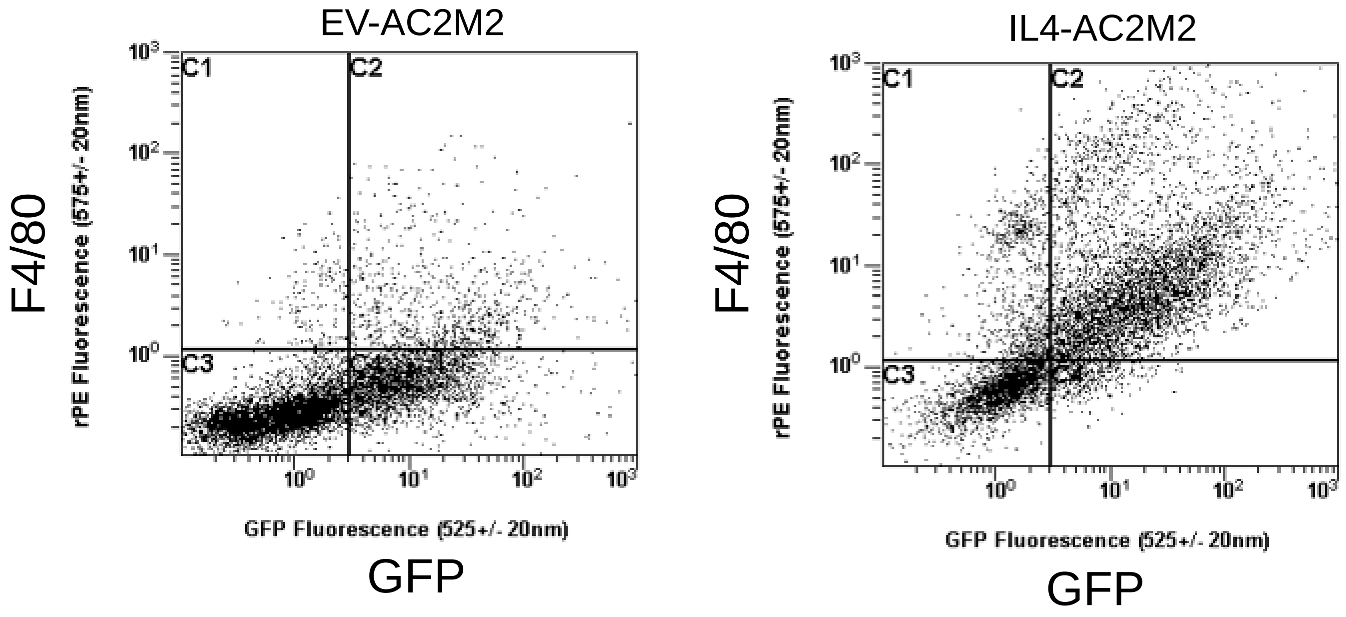

Supplementary Figure 9. Elevated macrophages and macrophage phagocytosis of tumor cells in IL-4 expressing tumor-bearing mammary glands. 7,500 GFP-expressing EV-AC2M2 or IL4-AC2M2 cells were engrafted into the fourth mammary glands of $Rag2^{-/-}$; $IL2R\gamma c^{-/-}$ mice and tumorigenesis was monitored. Control and IL-4 expressing tumor-bearing mammary glands were harvested on day 13 and 16, respectively, when tumors reached the same size. Single cell suspensions were prepared and stained with PE-conjugated F4/80 antibody to identify macrophages and the relative percentages of stained cells were determined by flow cytometry (n=8 for each cohort). There were significantly more macrophages in IL-4 expressing tumors and associated stroma relative to control ($P=0.0002$). Representative scatter plots show how the GFP^+ tumor cells were shifted upwards into the PE channel and become $F4/80^+$ GFP^+ double positive to a much greater extent in the IL4-AC2M2 tumors ($P<0.0001$), suggesting possible higher levels of macrophage phagocytosis of tumors cells or more macrophage and tumor cell interactions when IL-4 was present in the tumor microenvironment.

Supplementary Figure 10

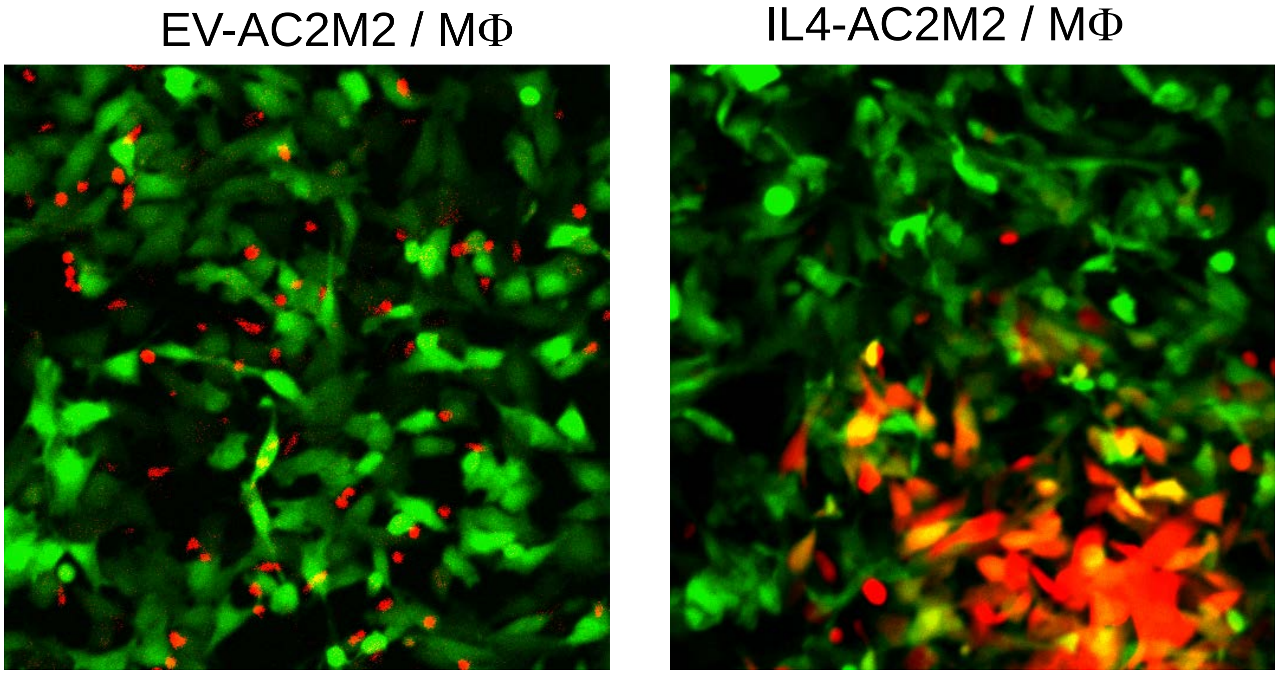

Supplementary Figure 10. Enhanced macrophage survival and phagocytic behavior in co-cultures with IL-4 expressing AC2M2 cells. Peritoneal macrophages (MΦ) were labeled with orange cell tracker dye (red), and then co-cultured with GFP-expressing EV-AC2M2 (EV) or IL4-AC2M2 (IL-4) tumor cells (green). Images were taken by spinning-disc microscopy after 24 hours of co-cultivation. Smaller macrophages predominate in co-cultures with control EV-AC2M2 cells. Larger macrophages as well as macrophage clusters (possible fused macrophages) were apparent in IL4-AC2M2 co-cultures. Yellow cells were also seen in IL4-AC2M2 co-cultures, indicative of macrophage-tumor cell fusion or phagocytosis events.
